# An Empirical Evaluation of Prompting Strategies for Large Language Models in Zero-Shot Clinical Natural Language Processing: Algorithm Development and Validation Study

**DOI:** 10.2196/55318

**Published:** 2024-04-08

**Authors:** Sonish Sivarajkumar, Mark Kelley, Alyssa Samolyk-Mazzanti, Shyam Visweswaran, Yanshan Wang

**Affiliations:** 1 Intelligent Systems Program University of Pittsburgh Pittsburgh, PA United States; 2 Department of Health Information Management University of Pittsburgh Pittsburgh, PA United States; 3 Department of Biomedical Informatics University of Pittsburgh Pittsburgh, PA United States

**Keywords:** large language model, LLM, LLMs, natural language processing, NLP, in-context learning, prompt engineering, evaluation, zero-shot, few shot, prompting, GPT, language model, language, models, machine learning, clinical data, clinical information, extraction, BARD, Gemini, LLaMA-2, heuristic, prompt, prompts, ensemble

## Abstract

**Background:**

Large language models (LLMs) have shown remarkable capabilities in natural language processing (NLP), especially in domains where labeled data are scarce or expensive, such as the clinical domain. However, to unlock the clinical knowledge hidden in these LLMs, we need to design effective prompts that can guide them to perform specific clinical NLP tasks without any task-specific training data. This is known as in-context learning, which is an art and science that requires understanding the strengths and weaknesses of different LLMs and prompt engineering approaches.

**Objective:**

The objective of this study is to assess the effectiveness of various prompt engineering techniques, including 2 newly introduced types—heuristic and ensemble prompts, for zero-shot and few-shot clinical information extraction using pretrained language models.

**Methods:**

This comprehensive experimental study evaluated different prompt types (simple prefix, simple cloze, chain of thought, anticipatory, heuristic, and ensemble) across 5 clinical NLP tasks: clinical sense disambiguation, biomedical evidence extraction, coreference resolution, medication status extraction, and medication attribute extraction. The performance of these prompts was assessed using 3 state-of-the-art language models: GPT-3.5 (OpenAI), Gemini (Google), and LLaMA-2 (Meta). The study contrasted zero-shot with few-shot prompting and explored the effectiveness of ensemble approaches.

**Results:**

The study revealed that task-specific prompt tailoring is vital for the high performance of LLMs for zero-shot clinical NLP. In clinical sense disambiguation, GPT-3.5 achieved an accuracy of 0.96 with heuristic prompts and 0.94 in biomedical evidence extraction. Heuristic prompts, alongside chain of thought prompts, were highly effective across tasks. Few-shot prompting improved performance in complex scenarios, and ensemble approaches capitalized on multiple prompt strengths. GPT-3.5 consistently outperformed Gemini and LLaMA-2 across tasks and prompt types.

**Conclusions:**

This study provides a rigorous evaluation of prompt engineering methodologies and introduces innovative techniques for clinical information extraction, demonstrating the potential of in-context learning in the clinical domain. These findings offer clear guidelines for future prompt-based clinical NLP research, facilitating engagement by non-NLP experts in clinical NLP advancements. To the best of our knowledge, this is one of the first works on the empirical evaluation of different prompt engineering approaches for clinical NLP in this era of generative artificial intelligence, and we hope that it will inspire and inform future research in this area.

## Introduction

Clinical information extraction (IE) is the task of identifying and extracting relevant information from clinical narratives, such as clinical notes, radiology reports, or pathology reports. Clinical IE has many applications in health care, such as improving diagnosis, treatment, and decision-making; facilitating clinical research; and enhancing patient care [1,2]. However, clinical IE faces several challenges, such as the scarcity and heterogeneity of annotated data, the complexity and variability of clinical language, and the need for domain knowledge and expertise.

Zero-shot IE is a promising paradigm that aims to overcome these challenges by leveraging large pretrained language models (LMs) that can perform IE tasks without any task-specific training data [3]. In-context learning is a framework for zero-shot and few-shot learning, where a large pretrained LM takes a context and directly decodes the output without any retraining or fine-tuning [4]. In-context learning relies on prompt engineering, which is the process of crafting informative and contextually relevant instructions or queries as inputs to LMs to guide their output for specific tasks [5]. The use of prompt engineering lies in its ability to leverage the powerful capabilities of large LMs (LLMs), such as GPT-3.5 (OpenAI) [6], Gemini (Google) [7], LLaMA-2 (Meta) [8], even in scenarios where limited or no task-specific training data are available. In clinical natural language processing (NLP), where labeled data sets tend to be scarce, expensive, and time-consuming to create, splintered across institutions, and constrained by data use agreements, prompt engineering becomes even more crucial to unlock the potential of state-of-the-art LLMs for clinical NLP tasks.

While prompt engineering has been widely explored for general NLP tasks, its application and impact in clinical NLP remain relatively unexplored. Most of the existing literature on prompt engineering in the health care domain focuses on biomedical NLP tasks rather than clinical NLP tasks that involve processing real-world clinical notes. For instance, Chen et al [9] used a fixed template as the prompt to measure the performance of LLMs on biomedical NLP tasks but did not investigate different kinds of prompting methods. Wang et al [10] gave a comprehensive survey of prompt engineering for health care NLP applications such as question-answering systems, text summarization, and machine translation. However, they did not compare and evaluate different types of prompts for specific clinical NLP tasks and how the performance varies across different LLMs. There is a lack of systematic and comprehensive studies on how to engineer prompts for clinical NLP tasks, and the existing literature predominantly focuses on general NLP problems. This creates a notable gap in the research, warranting a dedicated investigation into the design and development of effective prompts specifically for clinical NLP. Currently, researchers in the field lack a comprehensive understanding of the types of prompts that exist, their relative effectiveness, and the challenges associated with their implementation in clinical settings.

The main research question and objectives of this study are to investigate how to engineer prompts for clinical NLP tasks, identify best practices, and address the challenges in this emerging field. By doing so, we aim to propose a guideline for future prompt-based clinical NLP studies. In this work, we present a comprehensive empirical evaluation study on prompt engineering for 5 diverse clinical NLP tasks, namely, clinical sense disambiguation, biomedical evidence extraction, coreference resolution, medication status extraction, and medication attribute extraction [11,12]. By systematically evaluating different types of prompts proposed in recent literature, including prefix [13], cloze [14], chain of thought [15], and anticipatory prompts [16], we gain insights into their performance and suitability for each task. Two new types of prompting approaches were also introduced: (1) heuristic prompts and (2) ensemble prompts. The rationale behind these novel prompts is to leverage the existing knowledge and expertise in rule-based NLP, which has been prominent and has shown significant results in the clinical domain [17]. We hypothesize that heuristic prompts, which are based on rules derived from domain knowledge and linguistic patterns, can capture the salient features and constraints of the clinical IE tasks. We also conjecture that ensemble prompts, which are composed of multiple types of prompts, can benefit from the complementary strengths and mitigate the weaknesses of each individual prompt.

One of the key aspects of prompt engineering is the number of examples or shots that are provided to the model along with the prompt. Few-shot prompting is a technique that provides the model with a few examples of input-output pairs, while zero-shot prompting does not provide any examples [3,18]. By contrasting these strategies, we aim to shed light on the most efficient and effective ways to leverage prompt engineering in clinical NLP. Finally, we propose a prompt engineering framework to build and deploy zero-shot NLP models for the clinical domain. This study covers 3 state-of-the-art LMs, including GPT-3.5, Gemini, and LLaMA-2, to assess the generalizability of the findings across various models. This work yields novel insights and guidelines for prompt engineering specifically for clinical NLP tasks.

## Methods

### Tasks

We selected 5 distinct clinical NLP tasks representing diverse categories of natural language understanding: clinical sense disambiguation (text classification) [19], biomedical evidence extraction (named entity recognition) [20], coreference resolution [21], medication status extraction (named entity recognition+classification) [22], and medication attribute extraction (named entity recognition+relation extraction) [23]. Table 1 provides a succinct overview of each task, an example scenario, and the corresponding prompt type used for each task.

**Table 1:** Task descriptions.

Task	NLP^a^ task category	Description	Example prompt
Clinical sense disambiguation	Text classification	This task involves identifying the correct meaning of clinical abbreviations within a given context.	What is the meaning of the abbreviation CR^b^ in the context of cardiology?
Biomedical evidence extraction	Text extraction	In this task, interventions are extracted from biomedical abstracts.	Identify the psychological interventions in the given text?
Coreference resolution	Coreference resolution	The goal here is to identify all mentions in clinical text that refer to the same entity.	Identify the antecedent for the patient in the clinical note.
Medication status extraction	NER^c^+classification	This task involves identifying whether a medication is currently being taken, not taken, or unknown.	What is the current status of [24] in the treatment of [25]?
Medication attribute extraction	NER+RE^d^	The objective here is to identify specific attributes of a medication, such as dosage and frequency.	What is the recommended dosage of [26] for [27] and how often?

^a^NLP: natural language processing.

^b^CR: cardiac resuscitation.

^c^NER: named entity recognition.

^d^RE: relation extraction.

### Data Sets and Evaluation

The prompts were evaluated on 3 LLMs, GPT-3.5, Gemini, and LLaMA-2, under both zero-shot and few-shot prompting conditions, using precise experimental settings and parameters. To simplify the evaluation process and facilitate clear comparisons, we adopted accuracy as the sole evaluation metric for all tasks. Accuracy is defined as the proportion of correct outputs generated by the LLM for each task, using a resolver that maps the output to the label space. Table 2 shows the data sets and sample size for each clinical NLP task. The data sets are as follows:

- Clinical abbreviation sense inventories: This is a data set of clinical abbreviations, senses, and instances [28]. It contains 41 acronyms from 18,164 notes, along with their expanded forms and contexts. We used a randomly sampled subset from this data set for clinical sense disambiguation, coreference resolution, medication status extraction, and medication attribute extraction tasks (Table 2).
- Evidence-based medicine-NLP: This is a data set of evidence-based medicine annotations for NLP [29]. It contains 187 abstracts and 20 annotated abstracts, with interventions extracted from the text. We used this data set for the biomedical evidence extraction task.

**Table 2:** Evaluation data sets and samples for different tasks.

Task	Data set	Data set example	Samples
Clinical sense disambiguation	CASI^a^	The abbreviation “CR^b^” can refer to “cardiac resuscitation” or “computed radiography.”	11 acronyms from 55 notes
Biomedical evidence extraction	EBM^c^-NLP^d^	Identifying panic, avoidance, and agoraphobia (psychological interventions)	187 abstracts and 20 annotated abstracts
Coreference resolution	CASI	Resolving references to “the patient” or “the study” within a clinical trial report.	105 annotated examples
Medication status extraction	CASI	Identifying that a patient is currently taking insulin for diabetes.	105 annotated examples with 340 medication status pairs
Medication attribute extraction	CASI	Identifying dosage, frequency, and route of a medication for a patient.	105 annotated examples with 313 medications and 533 attributes

^a^CASI: clinical abbreviation sense inventories.

^b^CR: cardiac resuscitation.

^c^EBM: evidence-based medicine.

^d^NLP: natural language processing.

All experiments were carried out in different system settings. All GPT-3.5 experiments were conducted using the GPT-3.5 Turbo application programming interface as of the September 2023 update. The LLaMA-2 model was directly accessed for our experiments. Gemini was accessed using the Gemini application (previously BARD)—Google’s generative artificial intelligence conversational system. These varied system settings and access methods were taken into account to ensure the reliability and validity of our experimental results, given the differing architectures and capabilities of each LLM.

In evaluating the prompt-based approaches on GPT-3.5, Gemini, and LLaMA-2, we have also incorporated traditional NLP baselines to provide a comprehensive understanding of the LLMs’ performance in a broader context. These baselines include well-established models such as Bidirectional Encoder Representations From Transformers (BERT) [30], Embeddings From Language Models (ELMO) [31], and PubMedBERT-Conditional Random Field (PubMedBERT-CRF) [32], which have previously set the standard in clinical NLP tasks. By comparing the outputs of LLMs against these baselines, we aim to offer a clear perspective on the advancements LLMs represent in the field. This comparative analysis is crucial for appreciating the extent to which prompt engineering techniques can leverage the inherent capabilities of LLMs, marking a significant evolution from traditional approaches to more dynamic and contextually aware methodologies in clinical NLP.

### Prompt Creation Process

**Figure 1:**
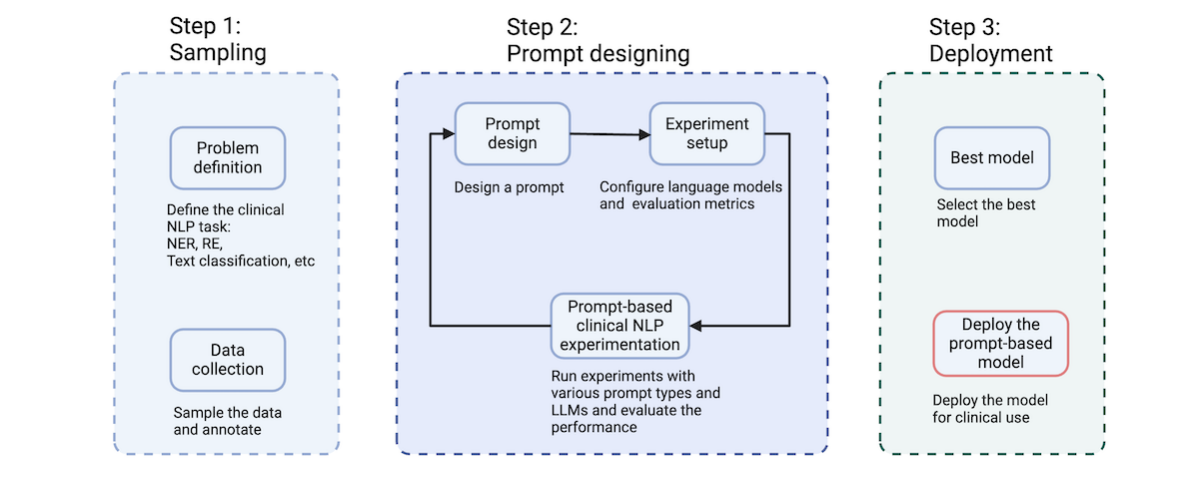
Iterative prompt design process: a schematic diagram of the iterative prompt creation process for clinical NLP tasks. The process consists of 3 steps: sampling, prompt designing, and deployment. The sampling step involves defining the task and collecting data and annotations. The prompt designing step involves creating and refining prompts using different types and language models. The deployment step involves selecting the best model and deploying the model for clinical use. LLM: large language model; NER: named entity recognition; NLP: natural language processing; RE: relation extraction.

A rigorous process was followed to create suitable prompts for each task. These prompts were carefully crafted to match the specific context and objectives of each task. There is no established method for prompt design and selection as of now. Therefore, we adopted an iterative approach where prompts, which are created by health care experts, go through a verification and improvement process in an iterative cycle, which involved design, experimentation, and evaluation, as depicted in Figure 1.

Figure 1 illustrates the 3 main steps of our prompt creation process: sampling, prompt designing, and deployment. In the sampling step (step 1), we defined the clinical NLP task (eg, named entity recognition, relation extraction, and text classification) and collected a sample of data and annotations as an evaluation for the task. In the prompt designing step (step 2), a prompt was designed for the task using one of the prompt types (eg, simple prefix prompt, simple cloze prompt, heuristic prompt, chain of thought prompt, question prompt, and anticipatory prompt). We also optionally performed few-shot prompting by providing some examples along with the prompt. The LLMs and the evaluation metrics for the experiment setup were then configured. We ran experiments with various prompt types and LLMs and evaluated their performance on the task. Based on the results, we refined or modified the prompt design until we achieved satisfactory performance or reached a limit. In the deployment step (step 3), the best prompt-based models were selected based on their performance metrics, and the model was deployed for the corresponding task.

### Prompt Engineering Techniques

#### Overview

Prompt engineering is the process of designing and creating prompts that elicit desired responses from LLMs. Prompts can be categorized into different types based on their structure, function, and complexity.

**Figure 2:**
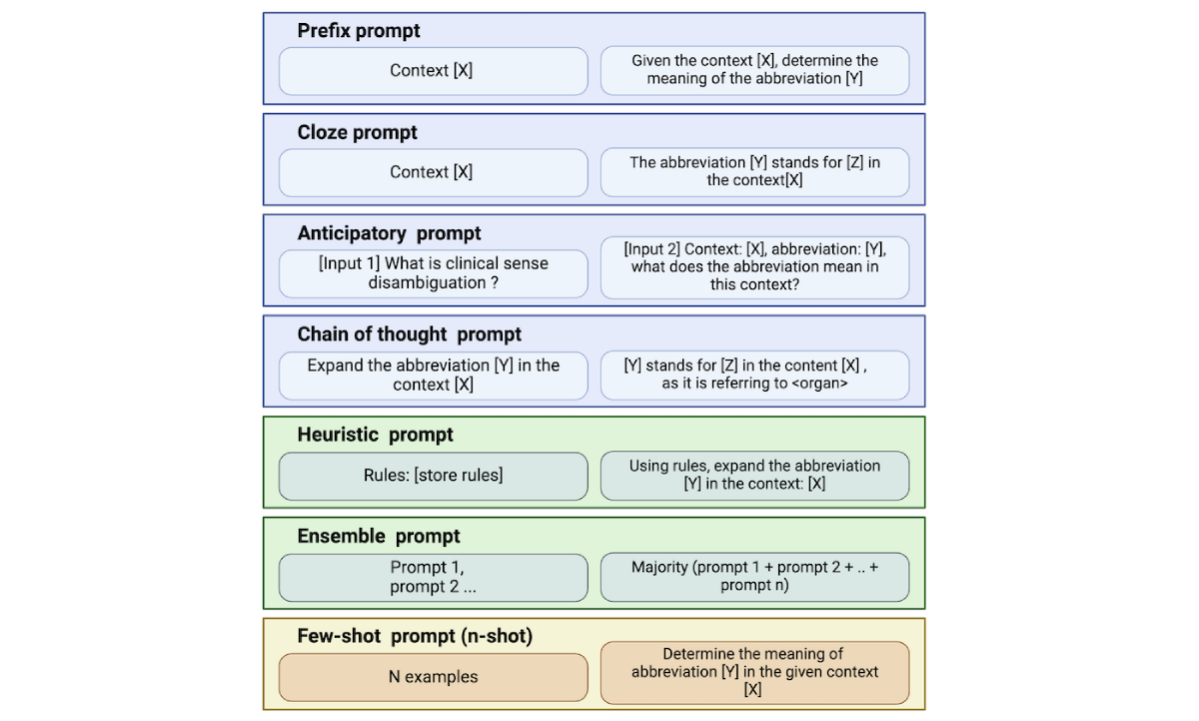
Types of prompts: examples of 7 types of prompts that we used to query the pretrained language model for different clinical information extraction tasks. [X]: context; [Y]: abbreviation; [Z]: expanded form.

Each prompt consists of a natural language query that is designed to elicit a specific response from the pretrained LLM. The prompts are categorized into 7 types, as illustrated in Figure 2 (all prompts have been included in Multimedia Appendix 1). Prefix prompts are the simplest type of prompts, which prepend a word or phrase indicating the type or format or tone of response for control and relevance. Cloze prompts are based on the idea of fill in the blank exercises, which create a masked token in the input text and ask the LLM to predict the missing word or phrase [3]. Anticipatory prompts are the prompts anticipating the next question or command based on experience or knowledge, guiding the conversation. Chain of thought prompting involves a series of intermediate natural language reasoning steps that lead to the final output [15].

In addition to the existing types of prompts, 2 new novel prompts were also designed: heuristic prompts and ensemble prompts, which will be discussed in the following sections.

#### Heuristic Prompts

Heuristic prompts are rule-based prompts that decompose complex queries into smaller, more manageable components for comprehensive answers. Adopting the principles of traditional rule-based NLP, which relies on manually crafted, rule-based algorithms for specific clinical NLP applications, we have integrated these concepts into our heuristic prompts approach. These prompts use a set of predefined rules to guide the LLM in expanding abbreviations within a given context. For instance, a heuristic prompt might use the rule that an abbreviation is typically capitalized, followed by a period, and preceded by an article or a noun. This approach contrasts with chain of thought prompts, which focus on elucidating the reasoning or logic behind an output. Instead, heuristic prompts leverage a series of predefined rules to direct the LLM in executing a specific task.

Mathematically, we can express a heuristic prompt as *H*(*x*), a function applied to an input sequence *x*. This function is defined as a series of rule-based transformations *T_i_*, where *i* indicates the specific rule applied. The output of this function, denoted as *y_H_*, is then:

y_H_=H(x)=T_n_(T_{n–1}_(... T_1_(x)))

Here, each transformation *T_i_* applies a specific heuristic rule to modify the input sequence, making it more suitable for processing by LLMs.

From an algorithmic standpoint, heuristic prompts are implemented by defining a set of rules *R={R_1_, R_2_, ..., R_m_*}. Each rule *R_j_* is a function that applies a specific heuristic criterion to an input token or sequence of tokens. Algorithmically, the heuristic prompting process can be summarized as follows:



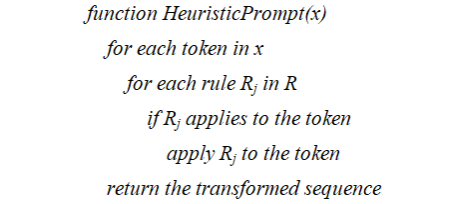



By merging the precision and specificity of traditional rule-based NLP methods with the advanced capabilities of LLMs, the heuristic prompts offer a robust and accurate system for clinical information processing and analysis.

#### Ensemble Prompts

Ensemble prompts are prompts that combine multiple prompts using majority voting for aggregated outputs. They use various types of prompts to generate multiple responses to the same input, subsequently selecting the most commonly occurring output as the final answer. For instance, an ensemble prompt might use 3 different prefix prompts, or a combination of other prompt types, to produce 3 potential expansions for an abbreviation. The most frequently appearing expansion is then chosen. For the sake of simplicity, we amalgamated the outputs from all 5 different prompt types using a majority voting approach.

Mathematically, consider a set of *m* different prompting methods *P*_1_, *P*_2_, ..., *P_m_* applied to the same input *x*. Each method generates an output *y_i_* for *i*=1,2, ..., *m*. The ensemble prompt’s output *y_E_* is then the mode of these outputs:

*y_E_*=mode (*y*_1_, *y*_2_, ..., *y_m_*)

Algorithmically, the ensemble prompting process is as follows:



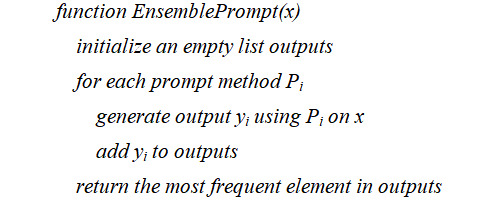



The rationale behind an ensemble prompt is that by integrating multiple types of prompts, we can use the strengths and counterbalance the weaknesses of each individual prompt, offering a robust and potentially more accurate response. Some prompts may be more effective for specific tasks or models, while others might be more resilient to noise or ambiguity. Majority voting allows us to choose the most likely correct or coherent output from the variety generated by different prompt types.

## Results

### Overview

In this section, we present the results of our experiments on prompt engineering for zero-shot clinical IE. Various prompt types were evaluated across 5 clinical NLP tasks, aiming to understand how different prompts influence the accuracy of different LLMs. Zero-shot and few-shot prompting strategies were also compared, exploring how the addition of context affects the model performance. Furthermore, we tested an ensemble approach that combines the outputs of different prompt types using majority voting. Finally, the impact of different LLMs on task performance was analyzed, and some interesting patterns were observed. Table 3 illustrates that different prompt types have different levels of effectiveness for different tasks and LLMs. We can also observe some general trends across the tasks and models.

**Table 3:** Performance comparison of different prompt types and language models.

Task and language model		Simple prefix	Simple cloze	Anticipatory	Heuristic	Chain of thought	Ensemble	Few shot
**Clinical sense disambiguation**								
	GPT-3.5	0.88	0.86	0.88	0.96^a^	0.9	0.9	0.82
	Gemini	0.76^b^	0.68	0.71	0.75	0.72	0.71	0.67
	LLaMA-2	0.88^b^	0.76	0.82	0.82	0.78	0.82	0.78
	BERT^c^ (from [33])	0.42	0.42	0.42	0.42	0.42	0.42	0.42
	ELMO^d^ (from [33])	0.55	0.55	0.55	0.55	0.55	0.55	0.55
**Biomedical evidence extraction**								
	GPT-3.5	0.92	0.82	0.88	0.94	0.94	0.88	0.96^a^
	Gemini	0.89	0.89	0.91^b^	0.9	0.91^b^	0.9	0.88
	LLaMA-2	0.85	0.88^b^	0.87	0.88^b^	0.87	0.88	0.86
	PubMedBERT-CRF^e^ (from [29])	0.35	0.35	0.35	0.35	0.35	0.35	0.35
**Coreference resolution**								
	GPT-3.5	0.78	0.6	0.74	0.94^a^	0.94^a^	0.74	0.74
	Gemini	0.69	0.81^b^	0.73	0.67	0.71	0.69	0.7
	LLaMA-2	0.8^b^	0.64	0.74	0.76	0.8^b^	0.78	0.68
	Toshniwal et al [34]	0.69	0.69	0.69	0.69	0.69	0.69	0.69
**Medication status extraction**								
	GPT-3.5	0.76^a^	0.72	0.75	0.74	0.73	0.75	0.72
	Gemini	0.67^b^	0.51	0.65	0.55	0.59	0.58	0.55
	LLaMA-2	0.58	0.48	0.52	0.64^b^	0.52	0.58	0.42
	ScispaCy [35]	0.52	0.52	0.52	0.52	0.52	0.52	0.52
**Medication attribute extraction**								
	GPT-3.5	0.88	0.84	0.9	0.96^a^	0.96^a^	0.9	0.96^a^
	Gemini	0.68	0.72	0.88^c^	0.7	0.74	0.76	0.88^b^
	LLaMA-2	0.6	0.66	0.58	0.66	0.72^b^	0.64	0.6
	ScispaCy	0.70	0.70	0.70	0.70	0.70	0.70	0.70

^a^Best performance on a task regardless of the model (ie, for each GPT-3.5 or Gemini or LLaMA-2 triple).

^b^Best performance for each model on a task.

^c^BERT: Bidirectional Encoder Representations From Transformers.

^d^ELMO: Embeddings From Language Models.

^e^PubMedBERT-CRF: PubMedBERT-Conditional Random Field.

### Prompt Optimization and Evaluation

For clinical sense disambiguation, the heuristic and prefix prompts consistently achieved the highest performance across all LLMs, significantly outperforming baselines such as BERT [30] and ELMO, with GPT-3.5 achieving an accuracy of 0.96, showcasing its advanced understanding of clinical context using appropriate prompting strategies. For biomedical evidence extraction, the heuristic and chain of thought prompts excelled across all LLMs in zero-shot setting. This indicates that these prompt types were able to provide enough information and constraints for the model to extract the evidence from the clinical note. GPT-3.5 achieved an accuracy of 0.94 with these prompt types, which was higher than any other model or prompt type combination. For coreference resolution, the chain of thought prompt type performed best among all prompt types with 2 LLMs—GPT-3.5 and LLaMA-2. This indicates that this prompt type was able to provide enough structure and logic for the model to resolve the coreference in the clinical note. GPT-3.5 displayed high accuracy with this prompt type, achieving an accuracy of 0.94. For medication status extraction, simple prefix and heuristic prompts yielded good results across all LLMs. These prompt types were able to provide enough introduction or rules for the model to extract the status of the medication in relation to the patient or condition. GPT-3.5 excelled with these prompt types, achieving an accuracy of 0.76 and 0.74, respectively. For medication attribute extraction, we found that the chain of thought and heuristic prompts were effective across all LLMs. These prompt types were able to provide enough reasoning or rules for the model to extract and label the attributes of medications from clinical notes. Anticipatory prompts, however, had the best accuracy for Gemini among all the prompts. GPT-3.5 achieved an accuracy of 0.96 with these prompt types, which was higher than any other model or prompt type combination.

**Figure 3:**
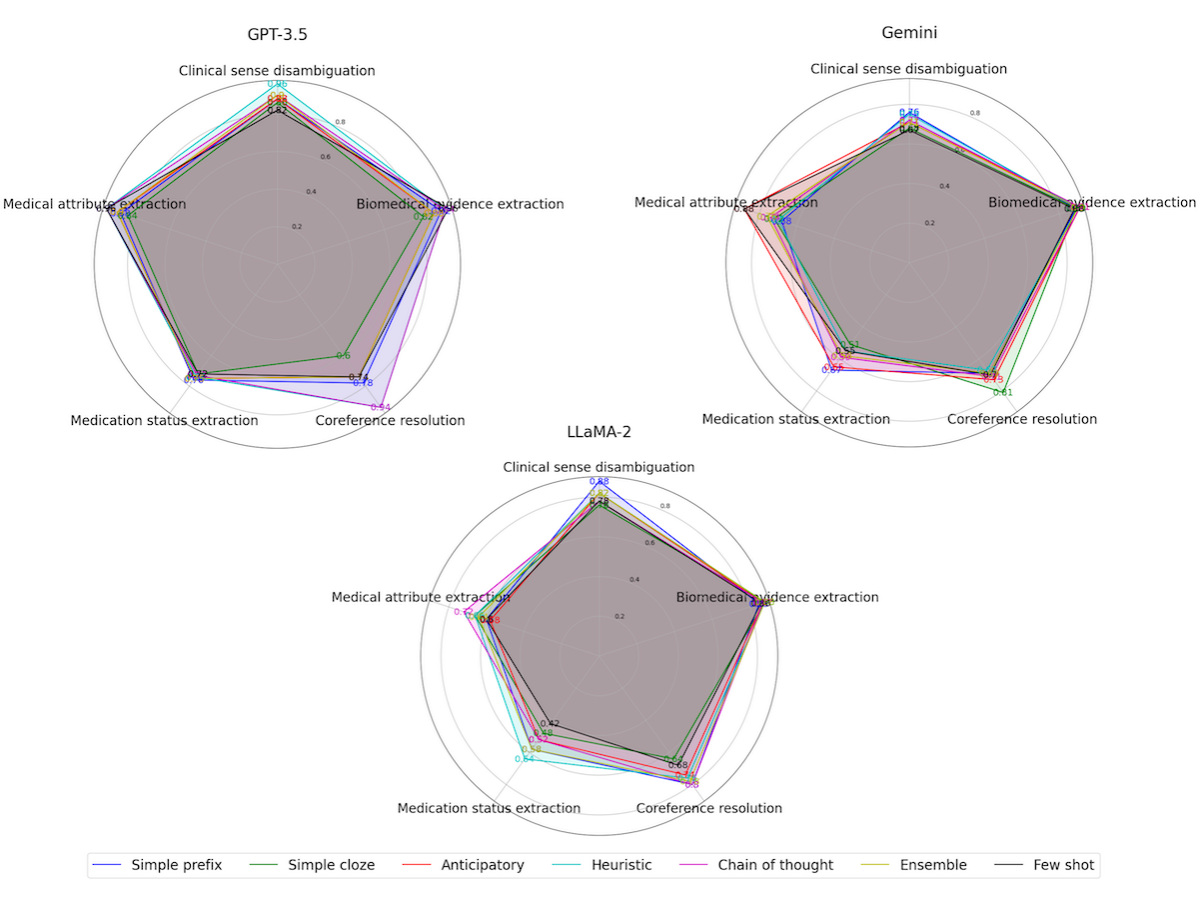
Graphical comparison of prompt types in the 5 clinical natural language processing tasks used in this study.

Thus, we can see that task-specific prompt tailoring is crucial for achieving high accuracy. Different tasks require different levels of information and constraints to guide the LLM to produce the desired output. The experiments show that heuristic, prefix, and chain of thought prompts are generally very effective for guiding the LLM to produce clear and unambiguous outputs. As shown in Figure 3, it is clear that GPT-3.5 is a superior and versatile LLM that can handle various clinical NLP tasks in zero-shot settings, outperforming other models in most cases.

Overall, the prompt-based approach has demonstrated remarkable superiority over traditional baseline models across all the 5 tasks. For clinical sense disambiguation, GPT-3.5’s heuristic prompts achieved a remarkable accuracy of 0.96, showcasing a notable improvement over baselines such as BERT (0.42) and ELMO (0.55). In biomedical evidence extraction, GPT-3.5 again set a high standard with an accuracy of 0.94 using heuristic prompts, far surpassing the baseline performance of PubMedBERT-CRF at 0.35. Coreference resolution saw GPT-3.5 reaching an accuracy of 0.94 with chain of thought prompts, eclipsing the performance of existing methods such as Toshniwal et al [34] (0.69). In medication status extraction, GPT-3.5 outperformed the baseline ScispaCy (0.52) with an accuracy of 0.76 using simple prefix prompts. Finally, for medication attribute extraction, GPT-3.5’s heuristic prompts achieved an impressive accuracy of 0.96, significantly higher than the ScispaCy baseline (0.70). These figures not only showcase the potential of LLMs in clinical settings but also set a foundation for future research to build upon, exploring even more sophisticated prompt engineering strategies and their implications for health care informatics.

### Zero-Shot Versus Few-Shot Prompting

The performance of zero-shot prompting and few-shot prompting strategies was compared for each clinical NLP task. The same prompt types and LLMs were used as in the previous experiments, but some context was added to the input in the form of examples or explanations. Two examples or explanations were used for each task (2-shot) depending on the complexity and variability of the task. Table 3 shows that few-shot prompting consistently improved the accuracy of all combinations for all tasks except for clinical sense disambiguation and medication attribute extraction, where some zero-shot prompt types performed better. We also observed some general trends across the tasks and models.

We found that few-shot prompting enhanced accuracy by providing limited context that aided complex scenario understanding. The improvement was more pronounced compared to simple cloze prompts, which had lower accuracy in most of the tasks. We also found that some zero-shot prompt types were very effective for certain tasks, even outperforming few-shot prompting. These prompt types used a rule-based or reasoning approach to generate sentences that contained definitions or examples of the target words or concepts, which helped the LLM to understand and match the context. For example, heuristic prompts achieved higher accuracy than few-shot prompting for clinical sense disambiguation and medication attribute extraction, while chain of thought prompts achieved higher accuracy than few-shot prompting for coreference resolution and medication attribute extraction. Alternatively, the clinical evidence extraction task likely benefits from additional context provided by few-shot examples, which can guide the model more effectively than the broader inferences made in zero-shot scenarios. This suggests that tasks requiring deeper contextual understanding might be better suited to few-shot learning approaches.

From these results, we can infer that LLMs can be effectively used for clinical NLP in a no-data scenario, where we do not have many publicly available data sets, by using appropriate zero-shot prompt types that guide the LLM to produce clear and unambiguous outputs. However, few-shot prompting can also improve the performance of LLMs by providing some context that helps the LLM to handle complex scenarios.

### Other Observations

#### Ensemble Approaches

We experimented with an ensemble approach by combining outputs from multiple prompts using majority voting. The ensemble approach was not the best-performing strategy for any of the tasks, but it was better than the low-performing prompts. The ensemble approach was able to benefit from the diversity and complementarity of different prompt types and avoid some of the pitfalls of individual prompts. For example, for clinical sense disambiguation, the ensemble approach achieved an accuracy of 0.9 with GPT-3.5, which was the second best–performing prompt type. Similarly, for medication attribute extraction, the ensemble approach achieved an accuracy of 0.9 with GPT-3.5 and 0.76 with Gemini, which were close to the best single prompt type (anticipatory). However, the ensemble approach also had some drawbacks, such as inconsistency and noise. For tasks that required more specific or consistent outputs, such as coreference resolution, the ensemble approach did not improve the accuracy over the best single prompt type and sometimes even decreased it. This suggests that the ensemble approach may introduce ambiguity for tasks that require more precise or coherent outputs.

While the ensemble approach aims to reduce the variance introduced by individual prompt idiosyncrasies, our specific implementation observed instances where the combination of diverse prompt types introduced additional complexity. This complexity occasionally manifested as inconsistency and noise in the outputs contrary to our objective of achieving higher performance. Future iterations of this approach may include refinement of the prompt selection process to enhance consistency and further reduce noise in the aggregated outputs.

#### Impact of LLMs

Variations in performance were observed among different LLMs (Table 3). We found that GPT-3.5 generally outperformed Gemini and LLaMA-2 on most tasks. This suggests that GPT-3.5 has a better generalization ability and can handle a variety of clinical NLP tasks with different prompt types. However, Gemini and LLaMA-2 also showed some advantages over GPT-3.5 on certain tasks and prompt types. For example, Gemini achieved the highest accuracy of 0.81 with simple cloze prompts and LLaMA-2 achieved the highest accuracy of 0.8 with simple prefix prompts for coreference resolution. This indicates that Gemini and LLaMA-2 may have some domain-specific knowledge that can benefit certain clinical NLP tasks for specific prompt types.

#### Persona Patterns

Persona patterns are a way of asking the LLM to act like a persona or a system that is relevant to the task or domain. For example, one can ask the LLM to “act as a clinical NLP expert.” This can help the LLM to generate outputs that are more appropriate and consistent with the persona or system. For example, one can use the following prompt for clinical sense disambiguation:

Act as a clinical NLP expert. Disambiguate the word “cold” in the following sentence: “She had a cold for three days.”

We experimented with persona patterns for different tasks and LLMs and found that they can improve the accuracy and quality of the outputs. Persona patterns can help the LLM to focus on the relevant information and constraints for the task and avoid generating outputs that are irrelevant or contradictory to the persona or system.

#### Randomness in Output

Most LLMs do not produce the output in the same format every time. There is inherent randomness in the outputs the LLMs produce. Hence, the prompts need to be specific in the way they are done for the task. Prompts are powerful when they are specific and if we use them in the right way.

Randomness in output can be beneficial or detrimental for different tasks and scenarios. In the clinical domain, randomness can introduce noise and errors in the outputs, which can make them less accurate and reliable for the users. For example, for tasks that involve extracting factual information, such as biomedical evidence extraction and medication status extraction, randomness can cause the LM to produce outputs that are inconsistent or contradictory with the input or context.

### Guidelines and Suggestions for Optimal Prompt Selection

In recognizing the evolving nature of clinical NLP, we expand our discussion to contemplate the adaptability of our recommended prompt types and LM combinations across a wider spectrum of clinical tasks and narratives. This speculative analysis aims to bridge the gap between our current findings and their applicability to unexplored clinical NLP challenges, setting a foundation for future research to validate and refine these recommendations. In this section, we synthesize the main findings from our experiments and offer some practical advice for prompt engineering for zero-shot and few-shot clinical IE. We propose the following steps for selecting optimal prompts for different tasks and scenarios:

The first step is to identify the type of clinical NLP task, which can be broadly categorized into three types: (1) classification, (2) extraction, and (3) resolution. Classification tasks involve assigning a label or category to a word, phrase, or sentence in a clinical note, such as clinical sense disambiguation or medication status extraction. Extraction tasks involve identifying and extracting relevant information from a clinical note, such as biomedical evidence extraction or medication attribute extraction. Resolution tasks involve linking or matching entities or concepts in a clinical note, such as coreference resolution.

The second step is to choose the prompt type that is most suitable for the task type. We found that different prompt types have different strengths and weaknesses for different task types, depending on the level of information and constraints they provide to the LLM. Table 4 summarizes our findings and recommendations for optimal prompt selection for each task type.

The third step is to choose the LLM that is most compatible with the chosen prompt type. We found that different LLMs have different capabilities and limitations for different prompt types, depending on their generalization ability and domain-specific knowledge. Table 5 summarizes our findings and recommendations for optimal LLM selection for each prompt type.

The fourth step is to evaluate the performance of the chosen prompt type and LLM combination on the clinical NLP task using appropriate metrics such as accuracy, precision, recall, or *F*_1_-score. If the performance is satisfactory, then the prompt engineering process is complete. If not, then the process can be repeated by choosing a different prompt type or LLM or by modifying the existing prompt to improve its effectiveness.

**Table 4:** Optimal prompt types for different clinical natural language processing task types.

Task type	Prompt type
Classification	Heuristic or prefix
Extraction	Heuristic or chain of thought
Resolution	Chain of thought

**Table 5:** Optimal language models for different prompt types.

Prompt type	Language model
Heuristic	GPT-3.5
Prefix	GPT-3.5 or LLaMA-2
Cloze	Gemini or LLaMA-2
Chain of thought	GPT-3.5
Anticipatory	Gemini

## Discussion

### Principal Findings

In this paper, we have presented a novel approach to zero-shot and few-shot clinical IE using prompt engineering. Various prompt types were evaluated across 5 clinical NLP tasks: clinical sense disambiguation, biomedical evidence extraction, coreference resolution, medication status extraction, and medication attribute extraction. The performance of different LLMs, GPT-3.5, Gemini, and LLaMA-2, was also compared. Our main findings are as follows:

1. Task-specific prompt tailoring is crucial for achieving high accuracy. Different tasks require different levels of information and constraints to guide the LLM to produce the desired output. Therefore, it is important to design prompts that are relevant and specific to the task at hand and avoid using generic or vague prompts that may confuse the model or lead to erroneous outputs.
2. Heuristic prompts are generally very effective for guiding the LLM to produce clear and unambiguous outputs. These prompts use a rule-based approach to generate sentences that contain definitions or examples of the target words or concepts, which help the model to understand and match the context. Heuristic prompts are especially useful for tasks that involve disambiguation, extraction, or classification of entities or relations.
3. Chain of thought prompts are also effective for guiding the LLM to produce logical and coherent outputs. These prompts use a multistep approach to generate sentences that contain a series of questions and answers that resolve the task in the context. Chain of thought prompts are especially useful for tasks that involve reasoning, inference, or coreference resolution.
4. Few-shot prompting can improve the performance of LLMs by providing some context that helps the model to handle complex scenarios. Few-shot prompting can be done by adding some examples or explanations to the input depending on the complexity and variability of the task. Few-shot prompting can enhance accuracy by providing limited context that aids complex scenario understanding. The improvement is more pronounced compared to simple prefix and cloze prompts, which had lower accuracy in most of the tasks.
5. Ensemble approaches can also improve the performance of LLMs by combining outputs from multiple prompts using majority voting. Ensemble approaches can leverage the strengths of each prompt type and reduce the errors of individual prompts. Ensemble approaches are especially effective for tasks that require multiple types of information or reasoning, such as biomedical evidence extraction and medication attribute extraction.

It is noteworthy that context size has a significant impact on the performance of LLMs in zero-shot IE [36]. In the scope of this study, we have avoided the context size dependence on performance, as it is a complex issue that requires careful consideration.

This study serves as an initial exploration into the efficacy of prompt engineering in clinical NLP, providing foundational insights rather than exhaustive guidelines. Given the rapid advancements in generative artificial intelligence and the complexity of clinical narratives, we advocate for continuous empirical testing of these prompt strategies across diverse clinical tasks and data sets. This approach will not only validate the generalizability of our findings but also uncover new avenues for enhancing the accuracy and applicability of LLMs in clinical settings.

### Limitations

In this study, we primarily focused on exploring the capabilities and versatility of generative LLMs in the context of zero-shot and few-shot learning for clinical NLP tasks. Our approach also has some limitations that we acknowledge in this work. First, it relies on the quality and availability of pretrained LLMs, which may vary depending on the domain and task. As LLMs are rapidly evolving, some parts of the prompt engineering discipline may be timeless, while some parts may evolve and adapt over time as different capabilities of models evolve. Second, it requires a lot of experimentation and iteration to optimize prompts for different applications, which may be iterative and time-consuming. However, once optimal prompts are identified, the approach offers time savings in subsequent applications by reusing these prompts or making minor adjustments for similar tasks. We may not have explored all the possible combinations and variations of prompts that could potentially improve the performance of the clinical NLP tasks. Third, the LLMs do not release the details of the data set that they were trained on. Hence, the high accuracy could be because the models would have already seen the data during training and not because of the effectiveness of the prompts.

### Future Work

We plan to address these challenges and limitations in our future work. We aim to develop more systematic and automated methods for prompt design and evaluation, such as using prompt-tuning or meta-learning techniques. We also aim to incorporate more domain knowledge or external resources into the prompts or the LLMs, such as using ontologies, knowledge graphs, or databases. We also aim to incorporate more quality control or error correction mechanisms into the prompts or the LLMs, such as using adversarial examples, confidence scores, or human feedback.

### Conclusions

In this paper, we have benchmarked different prompt engineering techniques for both zero-shot and few-shot clinical NLP tasks. Two new types of prompts, heuristic and ensemble prompts, were also conceptualized and proposed. We have demonstrated that prompt engineering can enable the use of pretrained LMs for various clinical NLP tasks without requiring any fine-tuning or additional data. We have shown that task-specific prompt tailoring, heuristic prompts, chain of thought prompts, few-shot prompting, and ensemble approaches can improve the accuracy and quality of the outputs. We have also shown that GPT-3.5 is very adaptable and precise across all tasks and prompt types, while Gemini and LLaMA-2 may have some domain-specific advantages for certain tasks and prompt types.

We believe that a prompt-based approach has several benefits over existing methods for clinical IE. It reduces the cost and time in the initial phases of clinical NLP application development, where prompt-based methods offer a streamlined alternative to the conventional data preparation and model training processes. It is flexible and adaptable, as it can be applied to various clinical NLP tasks with different prompt types and LLMs. It is interpretable and explainable, as it uses natural language prompts that can be easily understood and modified by humans.

## Appendix

Multimedia Appendix 1 Prompts for clinical natural language processing tasks.

## Abbreviations

BERT: Bidirectional Encoder Representations From Transformers
ELMO: Embeddings From Language Models
IE: information extraction
LLM: large language model
LM: language model
NLP: natural language processing
PubMedBERT-CRF: PubMedBERT-Conditional Random Field
