# Supplementary material for: An Empirical Evaluation of Prompting Strategies for Large Language Models in Zero-Shot Clinical Natural Language Processing: Algorithm Development and Validation Study
Source: JMIR Med Inform. 2024 Apr 8;12:e55318. doi: 10.2196/55318 (PMC11036183; doi:10.2196/55318)
Supplement: Multimedia Appendix 1 [file medinform_v12i1e55318_app1.docx]

**Multimedia Appendix 1. Prompts for clinical natural language processing tasks.**

In the appendix, we present the prompts that we used for each of the five clinical NLP tasks and each of the five prompt types in our experiments (Table S1). The prompts are written in natural language and formatted according to the input specifications of each language model. The prompt types are: Prefix, Cloze, Anticipatory, Heuristic, and Chain of Thought. We used seven types of prompts in our experiments, but two of them were not independent prompt types. Ensemble prompt type was a combination of the outputs from all the other prompt types, using majority voting to decide the final output. Few-shot prompting was a variation of the other prompt types, where we added two examples(2-shot) to the input to provide some context to the model. The clinical NLP tasks are: Clinical Sense Disambiguation, Biomedical Evidence Extraction, Coreference Resolution, Medication Status Extraction, and Medication Attribute Extraction.

**Table S1.** Prompts for each task and prompt type combination.

| **Task** | **Prefix** | **Cloze** | **Anticipatory** | **Heuristic** | **Chain-of-thought** |
| --- | --- | --- | --- | --- | --- |
| Clinical Sense Disambiguation | Determine the meaning to the abbreviation <abbreviation> in the clinical note. Clinical note: | The abbreviation <abbreviation> stands for ___ in the clinical note. Clinical Note: | \| [Input 1:] what is clinical sense disambiguation? \| \| --- \| \| [Input 2:] how to determine what a clinical abbreviation means in a given context? \| \| [Input 3:] Context: <text>, Abbreviation: <abbreviation>, What does the abbreviation mean in this context? \| | \| [Input 1:] First, store these rules in memory: \| \| --- \| \| 1. If the terms "hemoglobin," "ekg," or "valve" appear in the text, "ab" likely stands for "atrioventricular", "avr" likely stands for "aortic valve replacement","av" likely stands for "atrioventricular","cvp" likely stands for "central venous pressure","cvs" likely stands for cardiovascular system","la" likely stands for "left atrial", and "sa" likely stands for "sinuatrial" \| \| 2. If the text is discussing cardiology, cardiovascular issues, or the hearts electrical system, "ab" likely stands for "atrioventricular", "avr" likely stands for "aortic valve replacement","av" likely stands for "atrioventricular","cvp" likely stands for "central venous pressure","cvs" likely stands for cardiovascular system","la" likely stands for "left atrial", and "sa" likely stands for "sinuatrial" \| \| 3. If the text is relating to the heart, heart disease, or any anatomy relating to the heart, "ab" likely stands for "atrioventricular", "avr" likely stands for "aortic valve replacement","av" likely stands for "atrioventricular","cvp" likely stands for "central venous pressure","cvs" likely stands for cardiovascular system","la" likely stands for "left atrial", and "sa" likely stands for "sinuatrial" \| \| [Input 2:] Using the stored rules, expand the abbreviation "<abbreviation>" in the text: “<text>" \| | \| EXAMPLE: Expand the abbreviation "<abbreviation>" in the clinical note. Clinical Note: \| \| --- \| \| ANSWER: "<abbreviation>" stands for "<expanded form>" in the text because the context is referring to cardiology and the heart. \| \| QUESTION: Expand the abbreviation "<abbreviation>" in the clinical note. Clinical Note. \| |
| Biomedical Evidence Extraction | Label each token in the clinical note that references an intervention. If the token is an intervention, specify the type of intervention as: 1=surgical, 2=physical, 3=pharmacological,4=educational,5=psychological,6=other,7=control | For each token in the clinical note below using white space tokenization, the token relates to a __ intervention. Your choices are: surgical, physical, pharmacological, educational, psychological, other type, control, or not an intervention. | \| [Input 1:] what is medical intervention.  [Input 2:] how to identify medical interventions: \| \| --- \| \| [Input 2:] What intervention category does each token in the clinical note belong to? Surgical (1), physical (2), pharmacological (3), educational (4), psychological (5), other (6), control (7), or none (0). Use whitespace tokenization. Clinical Note: \| | \| [Input 1:] First, store these rules in memory: \| \| --- \| \|  \|   [Input 2:] Rule 1: Review each token and decide if the token can be classified as a “clinical intervention,” . Rule 2: If the token is not an intervention, assign “0” to the token, Rule 3. If the token is an intervention, use the following sub-category to classify the type of intervention: 1 = surgical, 2 = physical, 3 = pharmacological, 4 = educational, 5 = psychological, 6 = other, 7 = control.  [Input 3:] Using the above rules, extract the medical interventions in the clinical note. Clinical Note: | \| EXAMPLE: Assign each token in the clinical note to an intervention category: surgical (1), physical (2), pharmacological (3), educational (4), psychological (5), other (6), control (7), or none (0). Use whitespace tokenization. Clinical Note: "Report from Pain Study . Patients with opioid use responded better to treatment . Predicted outcome with morphine or vicodin ." \| \| --- \| \| ANSWER: Tokenized text using whitespace tokenization: "Report" "from" "Pain" "Study" "." "Patients" "with" "opioid" "use" "responded" "better" "to" "treatment" "." Predicted" "outcome" "with" "morphine" "or" "vicodin" "." A study is educational so "Pain" and "Study" will be assigned "4." A treatment and drug names are pharmacological so "treatment" "morphine" and "vicodin" will be assigned "3." \| \| QUESTION: Assign each token in the clinical note to an intervention category: surgical (1), physical (2), pharmacological (3), educational (4), psychological (5), other (6), control (7), or none (0). Use whitespace tokenization. Clinical Note: \| |
| Coreference Resolution | Identify the antecedent for the pronoun <pronoun> in the clinical note. Clinical Note: | The pronoun <pronoun> refers to ___ in the clinical note. Clinical Note: | \| [Input 1:] what is coreference resolution? \| \| --- \| \| [Input 2:] what is an antecedent? \| \| [Input 3:] What is the antecedent for the pronoun <pronoun> in the clinical note? Clinical Note: \| | \| [Input 1:] First, store these rules in memory.provided rules: identify the pronoun mentioned in the task, locate the pronoun in the text, determine the noun or noun phrase that the pronoun refers to by considering the context.> \| \| --- \| \|  \| \| [Input 2:] Identify the pronoun <pronoun> in the clinical text.  [Input 3:]identify the reference for the pronoun in the clinical note. Clinical Note: \| | \| EXAMPLE” Identify the antecedent the pronoun "this" refers to in the clinical note. Clinical Note: "5. Zosyn. This was stopped today, _%#MMDD2003#%_. 6. Multivitamin one tablet p.o. q.d. 7. Lasix 80 mg p.o. q.d. 8. Paxil 40 mg p.o. q.d. 9. Bactrim SS one tablet p.o. Mondays and Thursdays. 10. Ranitidine 150 mg p.o. b.i.d. 11. Metoprolol 25 mg p.o. b.i.d. 12. Colace 100 mg p.o. b.i.d." \| \| --- \| \| ANSWER: The pronoun "this" is referring to "Zosyn" because it is the most recent noun or noun phrase mentioned previously. \| \| QUESTION: identify the antecedent the pronoun "[pronoun]" refers to in the clinical note. Clinical Note: \| |
| Medication Status Extraction | In the clinical note, extract the medication and it’s current status as active, discontinued, or neither. Clinical note: | The active medications in the text are ___, the discontinued medications are ___, and the medications that are neither active nor discontinued in the clinical note are ___. Clinical Note: | \| [Input 1:] how to identify contextual level information in a text? \| \| --- \| \| [Input 2:] For each medication mentioned in the given clinical note, is the current status “active,” “discontinued,” or “neither?” \| | \| [Input 1:] First, store these rules in memory: \| \| --- \| \| <10 rules> \| \|  \| \| [Input 2:] Given the clinical note, extract the medication and its current status as active, discontinued, or neither, using the rules mentioned above. Clinical Note: \| | \| EXAMPLE: Label any medications in the clinical note as active, discontinued, or neither. Text: "2. Prior major leg infection. 3. Penicillin allergy. PLANS: Add Fortaz to vancomycin and DC clindamycin. Will also give one dose of tobramycin. If we do another I&D or dressing change, it would be of value to do swab culture of the wound to see what is colonizing it superficially." \| \| --- \| \| ANSWER: 1. Penicillin: Neither (doesn't specifically mention active or discontinued, if it's an allergy it's labeled as neither), 2. Fortaz: Active (states it was added to vancomycin, adding indicates it's an active medication), 3. Vancomycin: Active (text states Fortaz was added to Vancomycin, adding indicates it's an active medication), 4. Clindamycin: Discontinued (DC stands for discontinued). \| \| QUESTION: Using the stored example, label any medications in the text as active, discontinued, or neither. Text: <text> \| |
| Medication Attribute Extraction | Extract the medications mentioned in the clinical note, and any attributes such as route, frequency, dosage, duration, or reason for prescribing if mentioned. Clinical note: | The medications in the clinical note are ___. The name, dosage, frequency, and reason of each medication is ___. Clinical Note: | \| [Input 1:] how to extract contextual level information in a text such as status about medications? \| \| --- \| \| [Input 2:] how to identify specific medication attributes, such as route, frequency, or duration? \| \| [Input 3:] In the given clinical note, for each medication mentioned, what are the attributes such as route, frequency, dosage, duration, or reason for prescription? Clinical note: \| | \| [Input 1:] Store these rules in memory: <10 rules> \| \| --- \| \| [Input 2:] First extract the medications mentioned in the clinical note. Second extract any attributes such as route, frequency, dosage, duration, or reason for prescribing. \| | \|  \| \| --- \| \| EXAMPLE: Identify any medications in the text and list the name and if applicable the dosage, frequency, and reason. Text: "The patient tells me that she was taking 325 mg of aspirin per day for three years for a transient ischemic attack before she had her AVR. Now she is off this and on Coumadin, although the AVR is said to be a tissue valve. She takes no other nonsteroidal anti-inflammatory drugs except occasional Aleve in the past." \| \| ANSWER: 1. aspirin 325 mg per day for three years for a transient ischemic attack, 2. Coumadin, 3. nonsteroidal anti-inflammatory drugs, 4. Aleve occasional. \| \| QUESTION: Using the stored example, identify any medications in the clinical note and list the name and if applicable the dosage, frequency, and reason. Clinical Note: \| |
